# Reduced Carrier Recombination in PbS - CuInS_2_ Quantum Dot Solar Cells

**DOI:** 10.1038/srep10626

**Published:** 2015-05-29

**Authors:** Zhenhua Sun, Gary Sitbon, Thomas Pons, Artem A. Bakulin, Zhuoying Chen

**Affiliations:** 1 LPEM, PSL Research University, ESPCI-ParisTech, 10 rue Vauquelin, F-75231 Paris Cedex 5, France; 2 CNRS, UMR 8213, F-75005 Paris, France; 3 Sorbonne Universités, UPMC Univ Paris 06, F-75005 Paris, France; 4 FOM Institute AMOLF, Science Park 104, Amsterdam 1098 XG, The Netherlands

## Abstract

Energy loss due to carrier recombination is among the major factors limiting the performance of TiO_2_/PbS colloidal quantum dot (QD) heterojunction solar cells. In this work, enhanced photocurrent is achieved by incorporating another type of hole-transporting QDs, Zn-doped CuInS_2_ (Zn-CIS) QDs into the PbS QD matrix. Binary QD solar cells exhibit a reduced charge recombination associated with the spatial charge separation between these two types of QDs. A ~30% increase in short-circuit current density and a ~20% increase in power conversion efficiency are observed in binary QD solar cells compared to cells built from PbS QDs only. In agreement with the charge transfer process identified through ultrafast pump/probe spectroscopy between these two QD components, transient photovoltage characteristics of single-component and binary QDs solar cells reveal longer carrier recombination time constants associated with the incorporation of Zn-CIS QDs. This work presents a straightforward, solution-processed method based on the incorporation of another QDs in the PbS QD matrix to control the carrier dynamics in colloidal QD materials and enhance solar cell performance.

Solution synthesized and processed inorganic semiconducting nanocrystals, known as colloidal nanocrystal quantum dots (QDs), are under intense investigation for various optoelectronic applications such as photodetectors^1^, field-effect transistors^2^,^3^, light-emitting diodes^4^,^5^ and solar cells^6^,^7^,^8^,^9^,^10^. In particular, colloidal QDs offer exciting opportunities for photovoltaics due to their size-tunable bandgap and the multiple excitation generation phenomenon, a mechanism by which the Shockley-Queisser limit can be potentially bypassed^7^,^8^. Intensive investigations have been carried out on solar cells built from a variety of QDs of compositions such as CdS^11^, CdSe^12^,^13^, PbSe^8^,^14^, and PbS^9^,^10^,^15^,^16^ in the quest for high-performance and low-cost photovoltaic devices. Different device configurations have been proposed, such as QD-sensitized^11^, QD-organic bulk heterojunction^12^, metal oxide/QD bi-layer heterojunction solar cells^15^, and QD bulk nano-heterojunction^14^,^17^. So far, metal oxide/QD bi-layer depleted heterojunction solar cell is among the most efficient systems allowing power conversion efficiency (PCE) as high as 8.92% for PbS QD solar cells^16^,^18^,^19^,^20^. Significant improvements on both materials aspects and device performance are however still necessary to promote these solar cells as a viable technology for our society.

One important issue hindering the progress of many third-generation solar cells is carrier recombination^21^,^22^. In metal oxide/QD heterojunction solar cells, carrier recombination happens not only at the donor-acceptor (TiO_2_/QD) interface but also inside the QD layer which has a typical thickness of a few hundred nanometers. Photogenerated charges need to travel across the entire QD active layer to be collected. During this process carrier recombination leads to photocurrent loss and thus, to inefficient solar cells^11^. Due to the large surface-to-volume ratio in colloidal QDs, there can be abundant surface states in QD materials acting as recombination centers during charge transport^18^,^23^,^24^,^25^,^26^,^27^,^28^,^29^.

Under this context, the possibility to separate electrons and holes in different areas of the active layer, for example by using a mixture of different QDs, can lead to a substantial suppression of the recombination rates. Towards this goal, the recently introduced bulk nano-heterojunction device configuration, where *p*-type PbS QDs in the active layer are blended together with *n*-type QDs of a different composition, allows for higher device efficiencies^14^,^17^. In these devices the energy levels of the two QD components form a “type II” alignment leading to charge separation. While the “type II” energy level alignment used in these works can lead to efficient initial charge separation, it makes devices sensitive to the availability of charge percolation pathways, the blend morphology or the use of anisotropic QDs. If percolation pathways are not available, the charge concentration may build up and promote recombination inside the isolated *n*-type QDs. In this work, we propose a different approach based on the partial spatial segregation of charge carriers to boost photocurrent. Instead of *n*-type QDs, here Zn-doped CuInS_2_ (Zn-CIS) QDs, *p*-type QDs of reduced toxicity compared to PbS, are incorporated into the PbS matrix of TiO_2_/PbS QD heterojunction solar cells. In this binary QD blend Zn-CIS QDs provide recombination “shelters” where only holes but not electrons from PbS are allowed to enter. Due to the proximity of the HOMO levels between these two QDs, subsequent hole transport after charge separation is possible either within the Zn-CIS QD network or back into the PbS host. Different volume fractions of Zn-CIS QDs in the PbS QD matrix were examined: a 10% (v/v) addition of Zn-CIS QDs can lead to a ~30% increase in short circuit current density (*J*_*sc*_), a ~20% increase in power conversion efficiency (PCE), and prolonged recombination time constants compared to solar cells built from PbS QDs only.

## Results and Discussion

**Figure 1:**
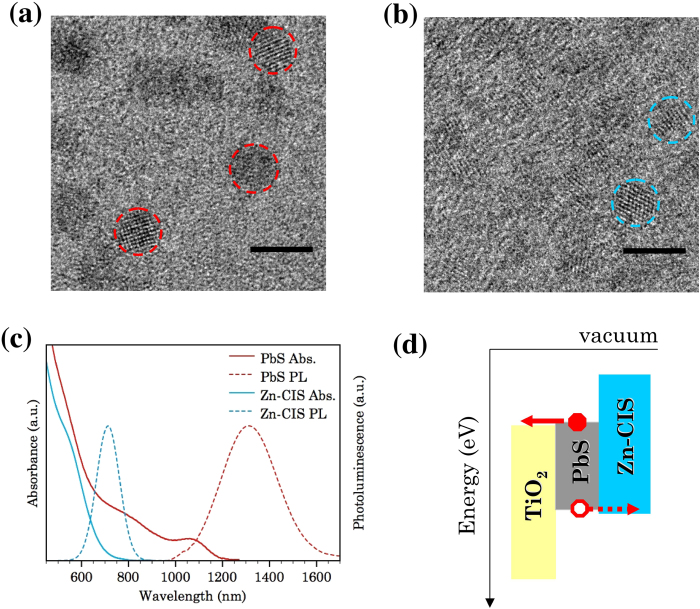
High-resolution TEM characterizations of PbS QDs (**a**) and Zn-CIS QDs (**b**). Scale bars correspond to 5 nm. Low-magnification TEM images are shown in the supporting information. (**c**) Thin film absorbance and photoluminescence spectra of PbS QDs and Zn-CIS QDs after MPA ligand-exchange. (**d**) Schematic (not to scale) of the HOMO and LUMO energy level alignment of the materials used in the active solar cell layer. The energy level alignment between PbS and Zn-CIS was estimated from cyclic voltammetry measurements.

Colloidal PbS and Zn-CIS nanocrystal QDs were synthesized by synthetic procedures reported previously^28^,^30^,^31^. As-synthesized QDs of both compositions are well-crystalline and of similar diameters between 4 and 5 nm (Fig. 1 and S1 in the supporting information). Zn-CIS QDs were used in this work instead of CIS QDs in order to reduce defects (such as cation vacancies) by the addition of Zn in the synthesis^32^. To allow electronic coupling between these two types of QDs in solar cells, the QD layer composed of either only PbS or a binary mixture of PbS and Zn-CIS QDs was deposited onto substrates via a layer-by-layer spin-coating and ligand-exchange procedure applying short-chain mercaptopropionic acid (MPA) ligands^6^,^28^,^33^. In both cases ligand-exchange was confirmed by Fourier transform infrared (FT-IR) spectroscopy which reveals new absorption signatures associated with the MPA molecules (Figure S2 and S3 in the supporting information). The optical bandgap of MPA-treated PbS QD thin films was estimated to be about 1.1 eV from the peak position of the first excitonic transition in the absorbance spectrum (Fig. 1c). The optical bandgap of MPA-treated Zn-CIS QD thin films was estimated to be about 1.9 eV from the onset of the absorbance spectrum^34^,^35^ (Fig. 1c). The HOMO and LUMO level alignments between these two types of QDs were estimated by the relative peak positions of the oxidation/reduction potentials from cyclic voltammetry (Figure S4): the HOMO level of Zn-CIS QDs lies slightly deeper (with reference to vacuum) than that of PbS QDs yet the difference (~0.2 eV) between them is relatively small considering the experimental error (±0.1 eV). By comparison, the LUMO level of Zn-CIS QDs is significantly higher-lying (~0.8 eV) than that of PbS QDs. This leads to a band alignment similar to that shown in Fig. 1d where charge transfer from PbS to Zn-CIS may be possible for holes but not for electrons. In addition, in such a binary system it is possible to have exciton energy transfer as well as charge transfer from Zn-CIS to PbS. The exciton energy transfer process can be beneficial in this case for photocurrent extraction as it allows excitons to sample more sites suitable for dissociation. Based on the electron transfer processes observed from CuInS_2_/ZnS core/shell QDs to TiO_2_^36^, similar charge transfer process may also be possible from Zn-CIS to PbS QDs considering the energy level offset between them.

**Figure 2:**
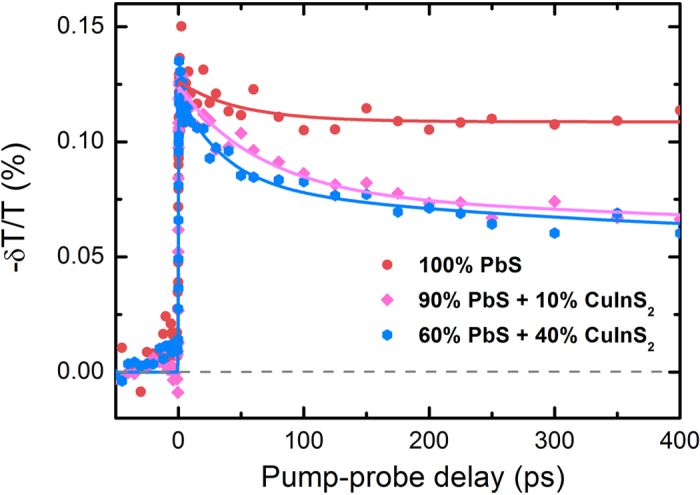
The results of visible-pump/IR-probe experiments performed on a PbS QD film and films containing a mixture of PbS and Zn-CIS QDs with different Zn-CIS volume fraction (10% (v/v) and 40% (v/v)). The pump photon energy was 1.55 eV (800 nm) and the probe photon energy was 0.31 eV (4000 nm).

Ultrafast visible-pump/mid-IR-probe spectroscopy was applied to characterize the charge photogeneration and early-time transport dynamics in single-component and binary QD thin films. Specifically, we compared the charge-induced absorption signature from PbS QDs in films containing only PbS QDs and films containing a binary mixture of PbS and Zn-CIS QDs (Fig. 2). QD films used in this study were deposited on CaF_2_ substrates by the same layer-by-layer spin-coating and MPA-ligand-exchange method^6^ as that used to fabricate solar cells. During ultrafast experiments, QD films were first irradiated with 800-nm wavelength pump pulses, exciting PbS QDs but *not* Zn-CIS QDs. The charge population was then probed by an IR beam with a wavelength of 4000 nm. We associate the response at 4000 nm with the 1s-1p electronic transition^28^. The signal shown in Fig. 2 reflects the absorption due to the existence of charges in photoexcited QDs. To avoid Auger recombination effects the pump energy was kept below 1 μJ/cm^2^ (the excitation fluence dependent transient absorption decays are shown in Figure S5). At such low fluxes the decay of the single-component PbS QD film at the picosecond-nanosecond timescale is negligible, which indicates slow (»1 ns) recombination in this material. By comparison, a clear decay is observable in binary QD samples, which can be attributed to either increased charge recombination or the fact of charge-transfer between these two QD components. As will be discussed below, increased photocurrent is observed in binary QD devices. The decay is therefore unlikely to be associated with recombination but is probably a signature of charge-transfer from PbS to Zn-CIS QDs taking place ~50 ps after the excitation. The decay is also unlikely to be associated with the doping-induced traps as its amplitude is very similar for the low and high doping samples. The absence of a significant further decay when the volume fraction of Zn-CIS QDs is increased from 10% to 40% is probably associated with the homogenous mixing of different QDs in the binary blend. At 10% volume fraction there is already in average more than one Zn-CIS QD neighboring each PbS QDs which guaranties the existence of charge transfer pathway. Therefore, increasing the number of Zn-CIS QDs does not substantially increase the probability of charge transfer events. The fact that the decay does not go to zero indicates that the IR absorption cross-section of charges at Zn-CIS QDs is smaller than those at PbS, but not negligible.

**Figure 3:**
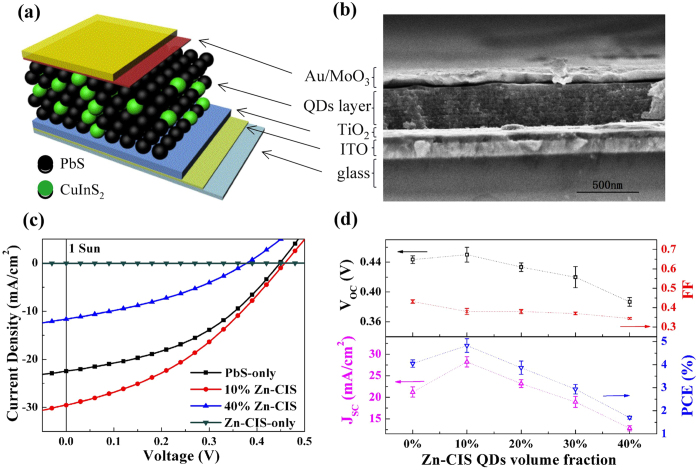
(**a**) Schematic of the bi-layer TiO_2_/QD heterojunction solar cell structure used in this work. The volume fraction of Zn-CIS QDs in the PbS QD layer was varied between 0%, 10%, 20%, 30%, 40%, and 100% (v/v). (**b**) Cross-section SEM image of a typical device. (**c**) Current-voltage (***J***-***V***) characteristics of four representative devices containing 0%, 10%, 40%, and 100% of Zn-CIS QDs in the PbS QD layer under 100 mW/cm^2^ simulated AM1.5G illumination. (**d**) The photovoltaic performance as a function of the volume fraction of Zn-CIS QDs incorporated. Each data point represents an average value based on ten solar cells fabricated and measured under identical conditions. *J*_*SC*_: short-circuit current; *V*_*OC*_: open-circuit voltage; FF: fill factor; PCE: power conversion efficiency.

We fabricated QD solar cells with either single-component QDs or a binary mixture of PbS and Zn-CIS QDs using the TiO_2_/QD depleted heterojunction device structure^37^ (Fig. 3a). The TiO_2_ layer was deposited onto indium tin oxide (ITO) substrates from a collosol solution followed by a high temperature crystallization process. The QD layer composed either of single component QDs (PbS or Zn-CIS) or binary PbS/Zn-CIS QDs was deposited on top of the TiO_2_ via a layer-by-layer spin-coating and ligand-exchange method with ten times of iteration (composed of ten sub-layers). The cross-section SEM image of a typical device (Fig. 3b) shows a ~50-nm-thick TiO_2_ layer and a ~250-nm-thick QD layer. Energy-dispersive X-ray (EDX) mapping coupled with SEM has been carried out on both planar and cross-sectional binary QD films revealing uniform presence of the two QD components inside the film ((Figure S6 to (S8). In addition, quantitative analysis from EDX spectroscopy confirms that the estimation of the volume fraction of each QD component in these hybrid QD films is accurate within +/−2% (Figure S6 to S8).

The photovoltaic performance of QD solar cells with different volume fraction of Zn-CIS QDs in the PbS QD layer was characterized under various illumination conditions. Four representative current-voltage (*J-V*) characteristics under 1 sun (AM 1.5 G) illumination from solar cells based on PbS-only, Zn-CIS-only, 10% and 40% Zn-CIS in PbS binary QDs are shown in Fig. 1c with the extracted photovoltaic parameters listed in Table 1. Remarkably, under 1-sun illumination, the 10% Zn-CIS binary QD device showed a short-circuit current (*J*_*SC*_) of 28.2 ± 1.2 mA/cm^2^, open-circuit voltage (*V*_*OC*_) of 0.45 ± 0.01 V, fill-factor (FF) of 0.38 ± 0.02 and power conversion efficiency (PCE) of 4.83 ± 0.29%. Compared to devices containing only PbS QDs, the main advantage of incorporating 10% Zn-CIS QDs into the PbS layer lies in the significant improvement of the *J*_*SC*_ (~30% increase) which leads to the overall efficiency enhancement of the solar cell. After experimenting different proportions of Zn-CIS QD incorporation (Fig. 3d), we found that 10% of Zn-CIS yielded an optimum result in this system and the incorporation of more Zn-CIS QDs brought no further benefits in photovoltaic properties. When the proportion of Zn-CIS QDs increases to 30%, we observed a reduction of *J*_*SC*_ and *V*_*OC*_ in the binary QD devices compared to PbS-only devices. Under low-intensity illumination (3% sun), further significant photovoltaic performance improvements associated with the presence of a moderate amount (10–20%) of Zn-CIS dots were observed in both *J*_*SC*_ and *V*_*OC*_ resulting in power conversion efficiencies as high as 7.7% (Table 1 and Figure S9). Such photovoltaic characteristics are important for emerging applications under dim light (e.g. indoor condition) to harvest energy.

**Figure 4:**
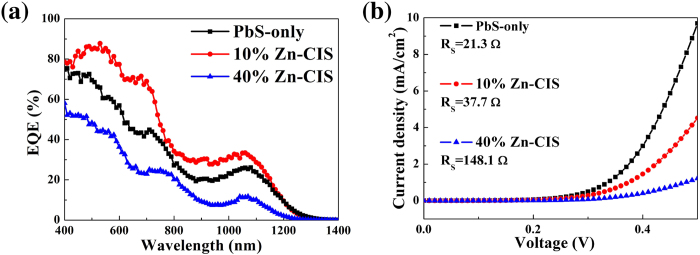
(**a**) The external quantum efficiency (EQE) of three representative TiO_2_/QD bi-layer heterojunction solar cells containing 0%, 10% and 40% volume fraction of Zn-CIS QDs in the PbS QD layer. (**b**) The dark current characteristics of these devices. The series resistances (*R*_*S*_) were extracted by fitting these dark current characteristics by a single diode model.

External quantum efficiencies (EQE) of three representative solar cells containing different volume fraction of Zn-CIS QDs are shown in Fig. 4. From these spectra, the effects on photocurrent associated with the incorporation of Zn-CIS dots can be seen over the entire spectrum. EQE spectra show very similar spectral signatures suggesting that the performance differences between PbS-only and binary-QD devices do not stem from the different light harvesting abilities of these two QD components. We also investigated solar cells based on Zn-CIS QDs only (without PbS QDs) under the same TiO_2_/QD heterojunction device structure (Fig. 3c). The very low *J*_*SC*_ obtained from these Zn-CIS-only devices may originate from the formation of a charge-blocking interface between TiO_2_ and Zn-CIS QDs. This also shows that the observed improvement of *J*_*SC*_ in 10% Zn-CIS/PbS binary QD devices does not arise from Zn-CIS QDs forming a better heterojunction with TiO_2_ compared to PbS QDs. It partly explains as well why binary QD solar cell performance starts to deteriorate when the volume fraction of Zn-CIS QDs further increases to ≥30%.

The dark current characteristics of PbS-only and binary QD solar cells suggest that the incorporation of Zn-CIS QDs leads to an increase of series resistance (Fig. 4b). Resistive losses are believed to be the major culprits leading to the reduced fill factors observed in binary QD devices^38^. The increased series resistance may come from the reduced hole mobility in Zn-CIS QDs compared to PbS and/or interfacial effects at TiO_2_ and metal contacts. Indeed, our field-effect mobility measurements on thin films containing PbS QDs, binary QDs, and Zn-CIS QDs reveal a reduction of hole mobility as the Zn-CIS QD volume fraction increases (Figure S10). It is known that both the polarity and the magnitude of the field-effect transistor (FET) mobility of colloidal QDs strongly depend on QD surface ligands, FET architecture, and the fabrication/measurement procedure (air-free or not)^39^,^40^,^41^. To correlate with our solar cell results we applied the same layer-by-layer MPA ligand-exchange procedure as that used in solar cells together with a simple bottom-gate (Si/SiO_2_) and bottom-contact (gold source/drain defined by lithography) architecture. FETs based on only PbS QDs exhibit *p*-type transport with a hole mobility of ~10^−4^ − 10^−3^ cm^2^V^−1^s^−1^. This is comparable with the reported mobility values of PbS-QD FETs with ethanedithiol ligands under a similar device architecture^39^. By comparison, FETs based on only Zn-CIS QDs exhibit similar hole transport characteristics but with a lower hole mobility of ~10^−6^ − 10^−5^ cm^2^V^−1^s^−1^. This is much lower than the mobility values reported in polycrystalline thin films of copper indium chalcogenides^42^ and may be related to factors such as the amount of QD surfaces (acting as hopping barriers), electronic coupling between QDs, charge trapping, and/or charge injections at the contacts of these Zn-CIS QD FETs. While the dark current and FET characteristics cannot explain the observed photovoltaic performance improvements in binary QD devices with a moderate amount of Zn-CIS dots (10–20%), they designate the rapidly increased series resistance as an important factor accounting for the degradation of photovoltaic performance in devices with a larger (≥30%) Zn-CIS QD concentration.

**Figure 5:**
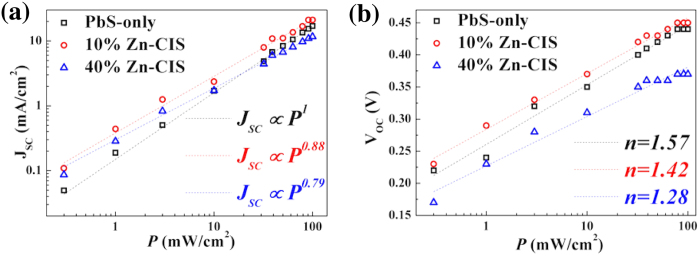
Light-intensity dependent *J*_*SC*_ and *V*_*OC*_ characteristics of the three representative devices containing 0%, 10% and 40% volume fraction of Zn-CIS QDs in the PbS QD layer. The experimental data (symbols) are fitted (dashed lines) using *J*_*SC*_ ∝ *P*^*A*^ and 
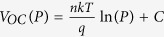
, where *k* is the Boltzmann constant, *T* is the temperature, *q* is the elemental charge, *A*, *n*, *C* are fitting parameters.

To gain insights in the mechanisms leading to the photovoltaic performance improvements in binary QD devices with a moderate volume fraction of Zn-CIS QDs, we studied how *J*_*SC*_ and *V*_*OC*_ depend on light intensity (*P*) in these devices (Fig. 5). For the *J*_*SC*_(*P*) relationship, if second-order bimolecular recombination is a dominant mechanism, *J*_*SC*_ saturates rapidly with increasing light intensity leading to a *J*_*SC*_ *∝ P*^*A*^ relationship with the power (*A*) approaching 0.5. This power value is however expected to be ∼1 if first-order monomolecular recombination processes (i.e. geminate or trap-assisted recombination) are dominating instead^13^,^43^. In reality, both bimolecular and monomolecular recombination mechanisms are present in solar cells leading to a power value between 0.5 and 1. Our *J*_*SC*_(*P*) results reveal a reduction of recombination parameter *A* from 1 in PbS-only solar cells to 0.88 in 10% Zn-CIS binary QD cells (Fig. 5a). A further reduction of the power value to 0.79 was observed in 40% Zn-CIS binary QD devices. This falls in line with ultrafast data (Fig. 2) and confirms that due to charge-transfer to Zn-CIS QDs, the relative strength of geminate or trap-assisted recombination in binary QD devices is reduced. As a result, bimolecular recombination tends to be more important in binary blend devices. The increased importance of bimolecular recombination in binary QD devices may also be associated with the higher lying LUMO of Zn-CIS QDs and the resultant reduced electron mobilities in these binary QD films.

For the *V*_*OC*_(*P*) dependence, the following relation^44^,^45^ has been proposed:
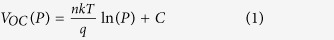
where *k* is the Boltzmann constant, *T* is the temperature, *q* is the elemental charge, *C* is a constant and *n* is the ideality factor. The ideality factor is expected to approach 2 when monomolecular recombination is the dominant mechanism while it is close to 1 if bimolecular recombination is dominating. Our results (Fig. 5b) reveal the reduction of the ideality factors along with the incorporation of Zn-CIS QDs. These *V*_*OC*_(*P*) trends also suggest that, along with the addition of Zn-CIS QDs in the PbS QD layer, the relative contribution of monomolecular recombination is reduced.

**Figure 6:**
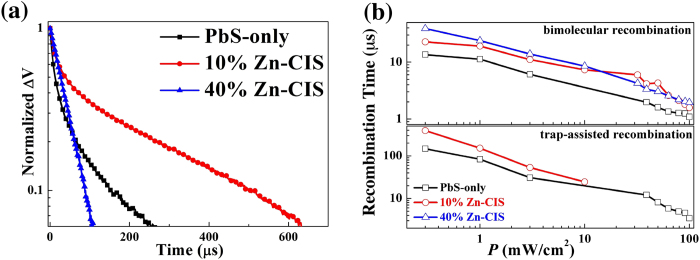
(**a**) Transient photovoltage characteristics of the three representative solar cells containing 0%, 10% and 40% volume fraction of Zn-CIS QDs in the PbS QD layer. In these measurements a laser pulse of 730 nm was used to generate a small perturbation ΔV on the steady *V*_*OC*_ induced by a static white light background illumination (intensity = 0.3 mW/cm^2^). The laser intensity was adjusted for the perturbation to be less than 5% of the *V*_*OC*_. Transient photovoltage data were normalized to the maximum value at the beginning of the decay for comparison. (**b**) Extracted recombination time versus background light intensity from the transient photovoltage decay in the devices used in (**a**). Time constants were extracted by fitting photovoltage transients with exponential functions. The decay dynamics of the 10% Zn-CIS device with *P* larger than 10 mW/cm^2^ and those of the 40% Zn-CIS device were fitted by mono-exponential function. The decay dynamics of the 10% Zn-CIS device with *P* less than 10 mW/cm^2^ and those of the PbS-only device were fitted by bi-exponential functions.

To probe more directly the long-time charge recombination kinetics, we used transient photovoltage techniques^13^,^46^,^47^ to characterize the same photovoltaic devices (Fig. 6). A laser pulse of 730 nm wavelength was shed onto PbS-only and binary QD solar cells and the following dynamics of the open-circuit voltage were monitored as a function of time. In addition to the laser pulse, a static white light was applied to induce a background photovoltage *V*_*OC*_. The laser pulse acts as a small perturbation to the white light creating a small magnitude of additional photovoltage (<5% of the background photovoltage). As the devices are under open-circuit condition, all carriers generated by the laser pulse eventually recombine. The decay of the transient photovoltage thus gives valuable information on carrier recombination.

Figure 6a shows the decay behaviors of the PbS-only and binary QD devices under a white light bias of 0.3 mW/cm^2^ intensity. The extracted recombination time constants from measurements with various background illumination intensities are summarized in Fig. 6b. When the background illumination intensity is relatively small (≤10 mW/cm^2^), the decay curves of the PbS-only and the 10% Zn-CIS device can be fitted by a superposition of two exponential functions suggesting two recombination mechanisms contributing with different time constants:^13^,^46^ Bimolecular recombination with ∼10 μs time constant (*t*_*bi*_) and trap-assisted recombination with a longer characteristic time constant (*t*_*tr*_). Both *t*_*bi*_ and *t*_*tr*_ are longer in the 10% Zn-CIS device compared to that in the PbS-only device indicating a general reduction of recombination rates upon the addition of Zn-CIS QDs. When the Zn-CIS QD volume fraction is further increased to 40%, the long time component disappears from the transient voltage decay curve, which can be fitted only by a mono-exponential function. This implies that a single recombination mechanism, namely the bimolecular recombination, dominates the 40% Zn-CIS device performance (Fig. 6b). When the background illumination increases to intensities larger than 10 mW/cm^2^, the decay curve of the 10% Zn-CIS device also transits to a mono-exponential regime. This is probably associated with the saturation of trap density of states, so that the bimolecular recombination increases more rapidly than trap-assisted recombination when the light intensity increases. By comparison, in PbS-only devices both recombination mechanisms are still clearly contributing to the global recombination dynamics. Overall, these transient photovoltage measurements confirm the presence of a reduced carrier recombination associated with the incorporation of Zn-CIS QDs.

It might seem surprising that such a small volume fraction (10%) of Zn-CIS QDs can lead to the observed improvement in the photovoltaic performance of PbS QD solar cells. However, in binary hard-spheres disordered mixtures, the onset of percolation, *i.e.* the threshold volume fraction for which infinite size clusters start to form, is only about 15%^48^. While sporadic Zn-CIS QDs in the PbS QD matrix can already provide temporal “shelters” against recombination during charge transport, if large clusters are available as expected for volume fractions close to the percolation threshold, holes can be safely transported within Zn-CIS QDs to the collecting electrode where chances of recombination is low. The reduced carrier recombination in binary QD solar cells thus contributes to the observed larger photocurrent and enhanced photovoltaic efficiency. In addition, based on the fast charge transfer (~50 ps) observed in ultrafast pump/probe spectroscopy, the formation of such a binary QD film can be a potential tool to counteract multiexciton Auger recombination (a major loss mechanism preventing carrier multiplication in colloidal quantum dot solar cells) as the biexciton lifetime of QDs of a similar diameter is typically on the order of 100 ps^49^,^50^. At a higher volume fraction of Zn-CIS QDs, if very large Zn-CIS clusters are formed, the number of energy and charge transfer events between Zn-CIS and PbS QDs may not further increase due to the increased distance between these two types of QDs especially for those Zn-CIS QDs inside a cluster. Therefore, at a higher Zn-CIS volume fraction the performance of binary QD solar cells become limited by other factors such as series resistance, inefficient electron transport, and the modification of TiO_2_/QD junction.

## Conclusions

In summary, we report a solution-processed approach to boost the performance of TiO_2_/PbS colloidal QD heterojunction solar cells by incorporating non-toxic Zn-doped CuInS_2_ (Zn-CIS) QDs inside the PbS QD matrix. Due to the proximity of the HOMO levels between PbS and Zn-CIS QDs, hole-transfer between them is permitted leading to spatial charge speparation inside the solar cell active layer. This charge transfer process was observed to occur within a few tens of picoseconds after photoexcitation by ultrafast optical spectroscopy. With 10% volume fraction of Zn-CIS QDs, the solar cell performance is significantly improved exhibiting a ~30% increase in *J*_*SC*_ and a ~20% increase in PCE under 1-Sun illumination. Under low-intensity illumination, the performance improvements in 10% Zn-CIS binary QD devices are even further improved, exhibiting enhancements in both *J*_*SC*_ and *V*_*OC*_. In agreement with illumination power density dependence experiments, transient photovoltage characteristics reveal longer recombination time constants in binary QD devices leading to the improved photocurrent and higher photovoltaic efficiency. Interestingly, the volume ratio of Zn-CIS QDs leading to the highest TiO_2_/binary-QD solar cell performance is very close to the volume fraction corresponding to the onset of percolation (~15%) expected in disordered binary hard-spheres systems in three dimensions. The present work provides a solution-processed “bottom-up” approach to achieve spatial charge separation and improved photovoltaic performance in metal oxide/QD heterojunction solar cells through the incorporation of another type of QDs into active layer.

## Methods

Synthesis. PbS quantum dots were synthesized by a procedure reported previously^28^,^30^ with a post-synthesis CdCl_2_ halide treatment^18^. Zn-CIS QDs were synthesized according to a reported procedure^6^. Detailed procedures on the QD synthesis are listed in the supporting information. The collosol for TiO_2_ was prepared by dissolving 2 ml titanium (IV) isopropoxide in 10 ml de-ionized (DI) water with 0.1 ml hydrochloric acid (37%). The mixture was stirred for 6 h at 80 °C then filtered by a 0.45-μm PVDF filter.

Material characterization. UV-Visible absorption spectra were recorded by a Varian Cary-5E spectrometer. The photoluminescence (PL) of PbS and Zn-CIS QD thin films was excited by a 515 nm (2–5 mW) continuous laser diode at room temperature in air and measured respectively by Ocean Optics NIRQUEST 256-2.5 (900–2500 nm) and HR4000 (400–900 nm) spectrometer coupled with an optical fiber. TEM and HRTEM images were obtained by a JEOL 2010 TEM (200 kV) equipped with a Gatan camera. Cross-section SEM images were acquired using FEI MAGELLAN 400 SEM with standard field emission gun source. Film thickness was measured using a profilometer (Veeco Dektak).

Ultrafast spectroscopy. The output of a regenerative 1 kHz Ti:Sapphire amplifier system (Coherent, Legend Elite Duo, 800 nm, 40 fs pulse duration, 7 mJ per pulse) was split into two parts. One part was attenuated and used as pump pulses. Another part was used to generate mid-IR (0.4 eV, 100 fs) probe pulses by pumping a commercial parametric amplifier with a difference frequency generation stage (HE TOPAS). Both beams were focused into ~100 um spot on the sample. To avoid Auger recombination effects the pump energy was kept below 1 mJ/cm^2^. The pump-probe transients were measured on a QD film deposited on top of CaF_2_ substrate. The probe and reference IR beams passed through the film and were detected by a nitrogen-cooled MCT detector array. The measurements were performed under N_2_ flow to avoid water vapor absorption of IR light and sample degradation.

Device fabrication. Indium tin oxide (ITO) substrates were cleaned in six sequential ultrasonic baths (for 10 minutes each) in a solution containing respectively 10% KOH in DI water, DI water, acetone, isopropanol and DI water. ITO substrates were then treated by O_2_ plasma for 10 minutes. A TiO_2_ layer of 50 nm thickness was then deposited on cleaned ITO substrates by spin-coating the collosol followed by a post-annealing at 500 °C for 30 minutes. The TiO_2_-coated ITO substrates were then transferred to an argon-filled glovebox for a layer-by-layer QD spin-coating and ligand exchange process^6^. The QDs solutions contain either PbS QDs (for PbS-only devices) or a uniform mixture of two QD solutions (PbS and Zn-CIS) of almost identical concentration under a certain volume ratio (for mixed QD devices). Before mixing we adjusted the solution concentration of these two types of QDs to be nearly identical through calibrating the thin film thickness achieved by the layer-by-layer deposition of single-component QDs with the same number of layer-by-layer iterations. The binary QD solution was then used in the layer-by-layer deposition procedure for mixed QD devices. For the layer-by-layer procedure: after spin-coating of each sub-layer, we dipped the sample into a methanol solution with 10% (v/v) 3-mercaptopropionic acid (MPA) for 30 s and then rinsed it with clean methanol, followed by a 2-minute solvent drying process on a hotplate at 50 °C. This process was repeated for 10 times to obtain a compact QD layer of 250 nm. QD samples were then taken out of the glovebox (involving an air exposure for abut 10–15 minutes) and loaded into a thermal evaporator. A 15-nm-thick MoO_3_ interfacial layer and a 100-nm-thick gold contact were then thermally evaporated in vacuum (<6 × 10^6^ mbar) through a shadow mask defining the device area to be 0.03 cm^2^. The devices were then encapsulated by epoxy inside a glovebox before electrical characterizations in air. The QD films for FETs, UV-Visible absorption and photoluminescence measurements were prepared by the fabrication procedure described above on different substrates (Si/SiO_2_(300 nm) for FETs and clean fused silica substrates for absorption and PL).

Device characterization. Solar cell current-voltage characteristics were measured by a Keithley 2612B source measurement unit (SMU). The devices were illuminated through the transparent substrate (ITO/glass) side by a class AAB (ASTM) ABET solar simulator (with an AM 1.5G filter) operated at 1 SUN. The light intensity was first calibrated by a calibrated Si reference solar cell. To obtain illumination of lower intensity a set of neutral density filters were used. For the EQE measurements, a monochromatic light beam was obtained from a white light source and an Oriel Cornerstone monochromator (and appropriate order sorting filters to eliminate higher order grating reflections). The light beam was chopped at 77 Hz. The monochromatic illumination was calibrated by a NIST-calibrated Si photodiode (for the 350 to 1000 nm range) and a NIST-calibrated Germanium photodiode (for the 1000–1400 nm range). In addition to the chopped monochromatic light, a set of LEDs were used to generate a static background white light of 45 mW/cm^2^ intensity to bias the device. The current response was measured through a Stanford Research systems SR570 low-noise current preamplifier and a SR810 DSP lock-in amplifier.

For transient photovoltage measurements, a temperature-controlled laser diode emitting at 730 nm was used together with a Agilent 33120A function/arbitrary waveform generator to generate laser pulses with a rise and fall time less than 100ns. The aforementioned ABET solar simulator together with various neutral density filters were used to supply a static background white light of different intensities. Transient photovoltage was amplified by the Stanford Research systems SR560 low-noise voltage preamplifier with an input impedance of 100 MΩ and then recorded by a Tektronix digital oscilloscope (DPO2024B) with input impedance of 1 MΩ. Field-effect transistor characterizations were performed in a probe station together with two Keithley 2400 SMUs under argon atmosphere. There was a brief air exposure (of a few minutes) for the FETs after they were taken out of a glovebox and before they are loaded inside an argon-filled test chamber.

## Additional Information

**How to cite this article**: Sun, Z. *et al.* Reduced Carrier Recombination in PbS - CuInS_2_ Quantum Dot Solar Cells. *Sci. Rep.* **5**, 10626; doi: 10.1038/srep10626 (2015).

## Supplementary Material

Supporting Information

## Figures and Tables

**Table 1:** Device performance summary for representative bi-layer TiO_2_/QD heterojunction solar cells containing 0%, 10%, and 40% (v/v) of Zn-CIS QDs in the PbS QD layer. Reported values are averaged on ten devices fabricated and measured under identical conditions.

Zn-CIS QDs (v/v %)	Illumina-tion	*J*_*SC*_(mA/cm^2^)	*V*_*OC*_(V)	Fill Factor	PCE (%)
0	1 SUN	21.20 ± 1.2	0.44 ± 0.01	0.43 ± 0.01	4.04 ± 0.16
	3% SUN	0.89 ± 0.11	0.32 ± 0.02	0.50 ± 0.01	4.76 ± 0.32
10	1 SUN	28.20 ± 1.2	0.45 ± 0.01	0.38 ± 0.02	4.83 ± 0.29
	3% SUN	1.54 ± 0.4	0.34 ± 0.01	0.45 ± 0.06	7.74 ± 1.37
40	1 SUN	12.80 ± 0.6	0.38 ± 0.01	0.34 ± 0.01	1.7 ± 0.06
	3% SUN	0.66 ± 0.01	0.32 ± 0.02	0.42 ± 0.01	2.86 ± 0.18
