# Supplementary material for: Reduced Carrier Recombination in PbS - CuInS2 Quantum Dot Solar Cells
Source: Sci Rep. 2015 May 29;5:10626. doi: 10.1038/srep10626 (PMC4448528; doi:10.1038/srep10626)
Supplement: Supporting Information [file srep10626-s1.pdf]

Supplementary Information

Reduced Carrier Recombination in PbS - $CuInS_2$ Quantum Dot Solar Cells

Zhenhua Sun,[1,2,3] Gary Sitbon,[1,2,3] Thomas Pons,[1,2,3] Artem A. Bakulin,[4] and Zhuoying Chen[1,2,3,*]

1. LPEM, PSL Research University, ESPCI-ParisTech, 10 rue Vauquelin, F-75231 Paris Cedex 5, France
2. CNRS, UMR 8213, F-75005 Paris, France
3. Sorbonne Universités, UPMC Univ Paris 06, F-75005 Paris, France
4. FOM Institute AMOLF, Science Park 104, Amsterdam 1098 XG, The Netherlands

* Address correspondence: zhuoying.chen@espci.fr

Table of Contents

I. Transmission electron microscopy of PbS and Zn-CIS QDs

II. Fourier transform infrared (FT-IR) spectroscopy

III. Cyclic voltammetry of PbS and Zn-CIS QDs

IV. The excitation fluence dependent transient absorption decays

V. Energy-dispersive X-ray (EDX)/SEM analysis of binary QD thin films

VI. Current-voltage (*J*-*V*) characteristics of TiO_2/QD bi-layer heterojunction solar cells containing different volume fraction of Zn-CIS QDs in the PbS QD layer under 3% SUN illumination

VII. Bottom-gate/bottom-contact field-effect transistor transfer characteristics of PbS QDs films containing different volume fractions of Zn-CIS QD deposited on Si/SiO_2 (300 nm) substrates.

VIII. Experimental details on synthesis

I. Transmission electron microscopy of PbS and Zn-CIS QDs

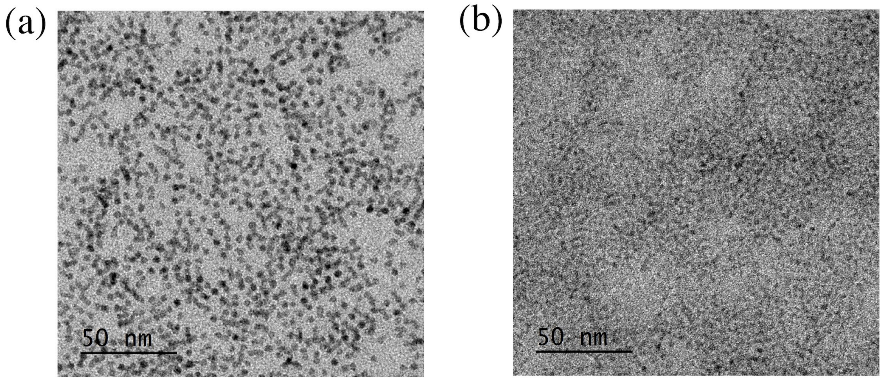

Figure S1. TEM characterizations of PbS QDs (a) and Zn-CIS QDs (b). Due to relativly lighter elements in the composition of Zn-CIS QDs compared to PbS, the TEM contrast of Zn-CIS QDs is weaker compared to that of PbS QDs.

II. Fourier transform infrared (FT-IR) spectroscopy

FT-IR spectroscopy was carried out on PbS and Zn-CIS QDs thin films before and after ligand exchanged by 3-mercaptopropionic acid (MPA) via a layer-by-layer spin-coating process (described in the experimental section). For PbS QDs (**Figure S2**), both oleic acid and MPA-capped QD films show carboxylate absorption signatures at 1400 - 1750 cm^{-1}. Upon ligand-exchange, the absorption intensity around 2900 cm^{-1} is significantly reduced, indicating a lost of CH stretching vibrations. This result agrees well with previous studies (K.S. Jeong *et al.*, ACS Nano, 2012, 6(1), 89-99) and reflects the exchange of the oleic acid by MPA.

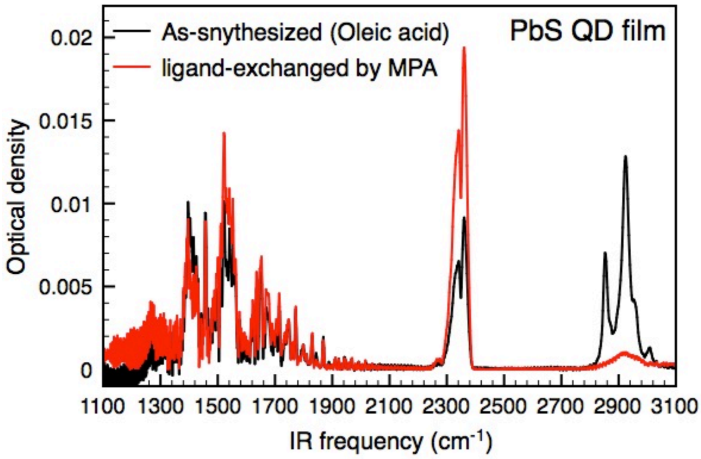

Figure S2. FT-IR absorbance of PbS QD thin films spin-coated on polished silicon substrates. These QDs films are either non-treated (black curve, capped by as-synthesized ligands of oleic acid) or ligand-exchanged by MPA via a layer-by-layer procedure (red curve). FT-IR absorbance was measured in air; the CO_2 in air leads to the absorption signature at about 2300 $cm^{-1}$and multiple narrow peaks in the 1600 - 1900 cm^{-1} region are associated with the residual water vapor absorption.

For Zn-CIS QDs (**Figure S3**), comparing QDs films with as-synthesized ligands (1-dodecanethiol) and films after MPA ligand-exchange, there is a clear difference in the absorption signatures in the vibrational fingerprint region between 1400 cm^{-1} and 1730 cm^{-1}. The absorption shared by both samples at about 1450 cm^{-1} originates from a superposition of C-H, and potentially S-H bending modes. The absorptions at 1550 - 1730 cm^{-1} are due to O-H bending and C=O stretches which are only present in MPA-capped QD films. This result reflects the exchange of the 1-dodecanethiol by MPA in these QD films.

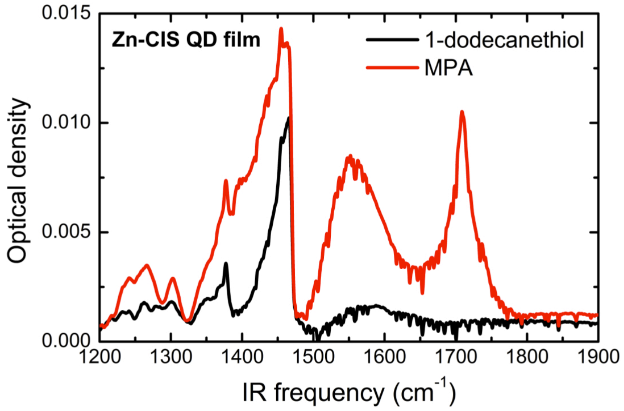

Figure S3. FT-IR absorbance of Zn-CIS QD thin films spin-coated on CaF_2 substrates. These QDs films are either non-treated (black curve, capped by as-synthesized ligands of 1-dodecanethiol) or ligand-exchanged by MPA via a layer-by-layer procedure (red curve). FT-IR absorbance was measured in vacuum.

III. Cyclic voltammetry of PbS and Zn-CIS QDs

Cyclic voltammograms were recorded under nitrogen atmosphere by a Potentiostat Galvanostat (Uniscan Instruments, PG581), with a circular gold disk (diameter = 3 mm) as the working electrode, a Pt wire as the counter-electrode, and Ag/$AgNO_3$ (Ag wires with 0.01 M $AgNO_3$ in anhydrous acetonitrile) as the anhydrous reference electrode. 0.1 M tetrabutylammonium hexafluorophosphate was dissolved in anhydrous acetonitrile and used as the supporting electrolyte. The reference electrode was calibrated by ferrocene/

ferrrocenium (Fc+/Fc) redox reaction. The samples used for cyclic voltammetry measurements were fabricated under inert atmosphere by coating QDs onto a 3-mm-diameter circular gold disk microelectrode (the working electrode). The QDs on this electrode also went through the ligand exchange procedure by its immersion into a solution of methanol containing 10% (v/v) of MPA for 30 s followed by subsequent solvent rinsing and drying. Multiple coating and ligand exchange iterations were performed in order to reach a compact QD films on the electrode.

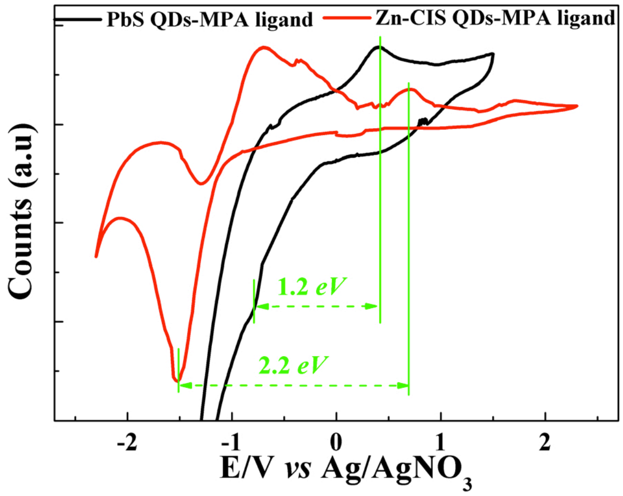

Figure S4. Cyclic voltammetry of MPA-capped PbS and Zn-CIS QDs.

IV. The excitation fluence dependent transient absorption decays

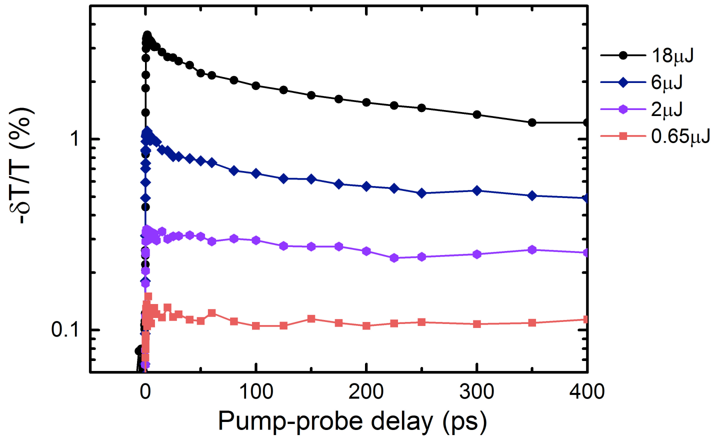

Figure S5. The excitation fluence dependent transient absorption decay observed in vis-pump/IR-probe experiments on PbS QD films. The pump photon energy was 1.55 eV and probe photon energy 0.31eV. The fast decay component, observed when using high (18 - 6 uJ) excitation energy, is associated with the Auger recombination of photogenerated species. When the pump energy drops below 2uJ, the transients become energy independent which

indicates that there is no more than one excitation per QD. The measurements presented in the paper have been performed using 0.65 uJ excitation energy.

V. Energy-dispersive X-ray (EDX)/SEM analysis of binary QD thin films

(1) Planar sample characterizations

The hybrid QD layer of the photovoltaic devices described in this work composes typically ten sub-layers which are deposited by ten times of layer-by-layer spin-coating and ligand-exchange process. The results of SEM imaging coupled with EDX elementary mapping and EDX spectroscopy from one sub-layer of hybrid QD film (40% volume fraction of Zn-CIS QDs in PbS QDs) on an ITO/glass substrate are shown below (Figure S6). EDX mapping reveals a uniform presence of Pb and Cu elements in the whole sample area and thus confirms a uniform mixing of the two QD components in one sub-layer.

Quantitative analysis from the EDX spectra of the same area (Figure S6 (d)) indicates an atomic percentage of Pb and Cu to be respectively 0.05% and 0.02%. As the QD film is composed of only two types of QDs (PbS and Zn-CIS), from the measured atomic (molar) ratio between these two QD components, combining their molar masses and densities, the volume fraction of Zn-CIS QDs in the QDs layer is calculated to be 40.6%. This result is in excellent agreement with the 40% volume fraction estimated from the formulation of binary QD solutions.

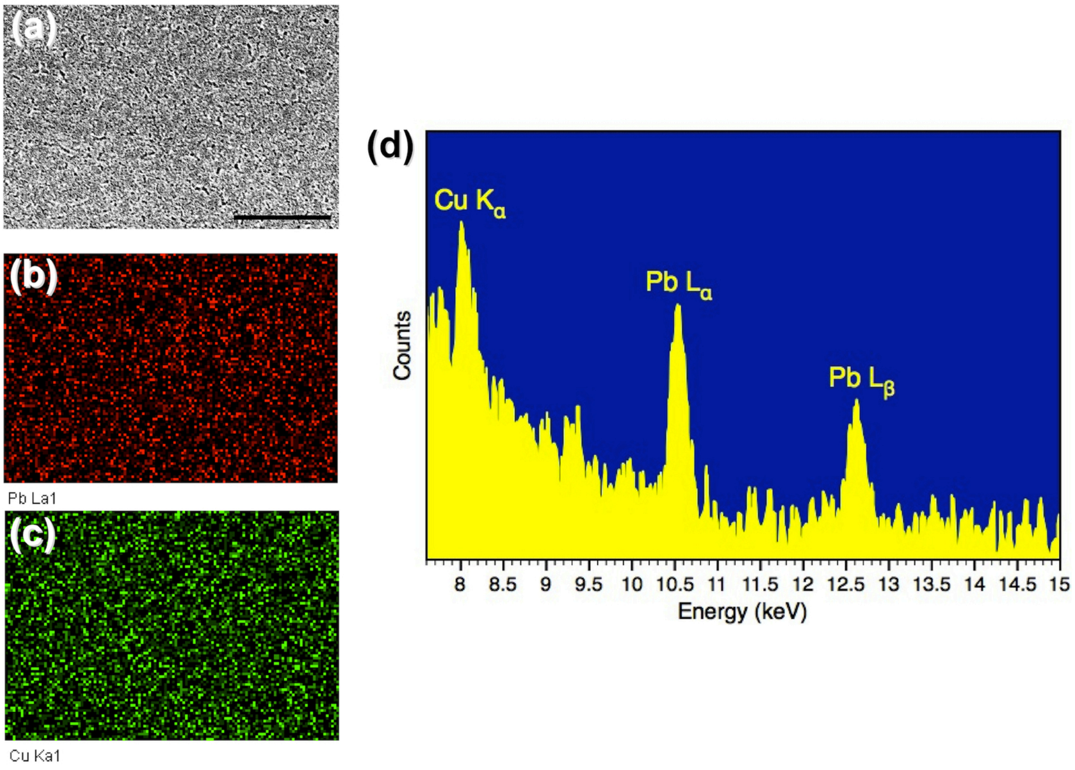

Figure S6. (a) SEM image of one sub-layer of hybrid QDs containing 40% of Zn-CIS QDs in PbS QDs deposited onto an ITO/glass substrate by spin-coating with ligand-exchange to MPA. The film thickness is about 25 nm. The scale bar corresponds to 300 nm; (b) and (c) EDX mapping of Pb (b) and Cu (c) elements on the same sample area of (a); (d) the EDX spectra detected from this area.

Note that it is difficult to perform the same quantitative analysis on one sub-layer of hybrid QD film with 10% of Zn-CIS QDs in PbS QDs due to the scarcity of Cu content. Therefore, we performed similar analysis on a thicker hybrid QD film (with ten sub-layers) with 10% volume fraction of Zn-CIS (Figure S7). EDX mapping also indicates a uniform mixing of the two QD components in this sample. Quantitative analysis from the EDX spectra of the same area (Figure S7 (d)) indicates an atomic percentage of Pb and Cu to be respectively 0.45% and 0.03%. From the measured atomic (molar) ratio between the two QD components, combining their molar masses and densities, the volume fraction of Zn-CIS QDs in the QDs layer is calculated to be 10.2%. This result is in excellent agreement with the 10% volume fraction estimated from the formulation of binary QD solutions.

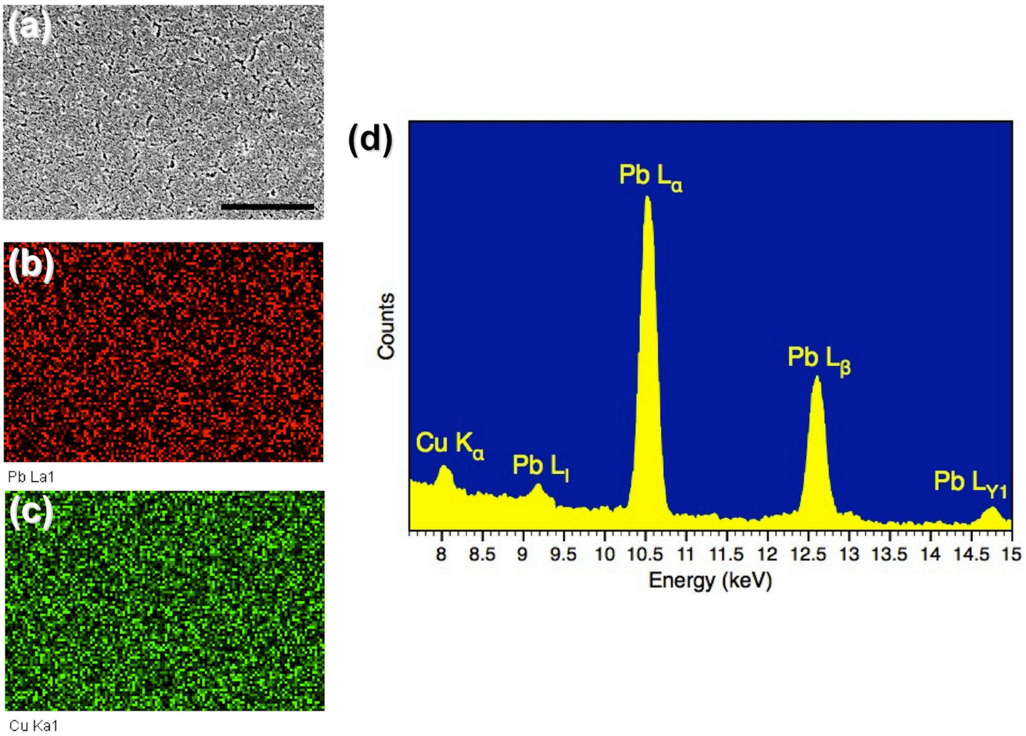

Figure S7. (a) SEM image of a film of hybrid QDs containing 10% of Zn-CIS QDs in PbS QDs deposited onto an ITO/glass substrate by layer-by-layer spin-coating and ligand-exchange process (with ten iterations). The scale bar corresponds to 300 nm; (b) and (c) EDX mapping of Pb (b) and Cu (c) elements on the same sample area of (a); (d) the EDX spectra detected from this area.

(2) Cross-sectional sample characterizations

Similar EDX mapping and spectroscopy analysis were carried out a hybrid QD film with 10% volume fraction of Zn-CIS deposited by a layer-by-layer process with ten iterations on a Si substrate (Figure S8). EDX mapping reveals a uniform presence of Pb and Cu elements along the thickness direction which indicates a uniform mixing of the two QD components. Quantitative analysis from the EDX spectra of the same area (Figure S8 (g)) indicates an atomic percentage of Pb and Cu to be respectively 4.84% and 0.26%. From the measured atomic (molar) ratio between the two QD components, combining their molar masses and densities, the volume fraction of Zn-CIS QDs is calculated to be 8.4%. This result is in agreement (within 2% error) with the 10% volume fraction estimated from the formulation of binary QD solutions.

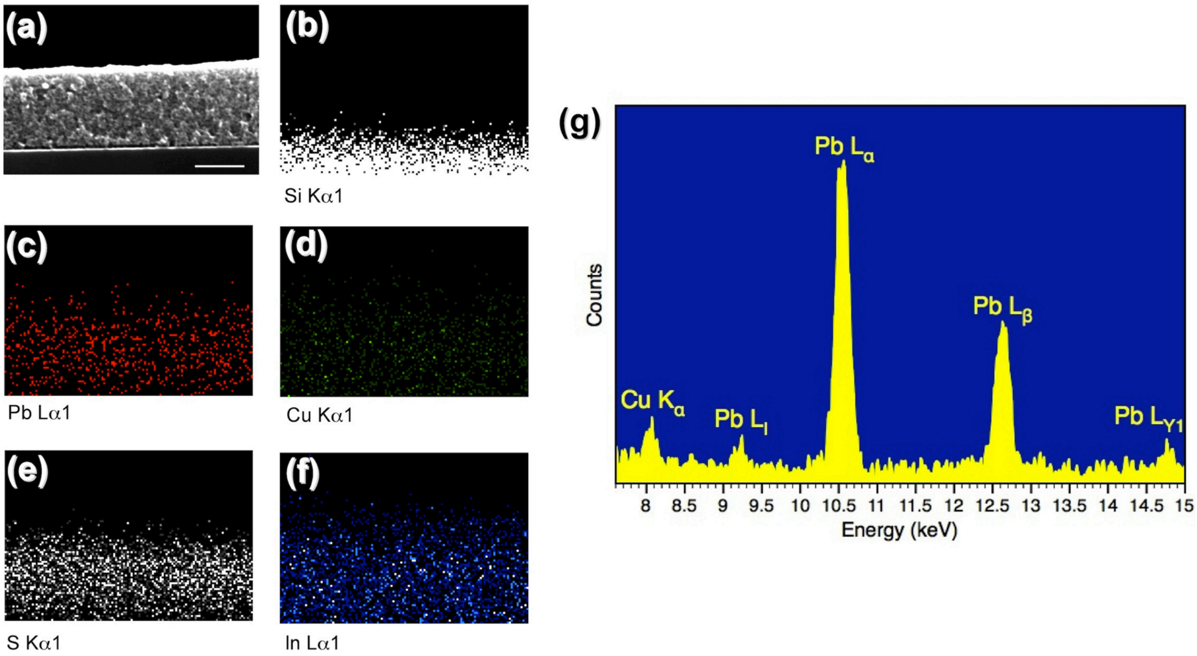

Figure S8. (a) SEM image of a cross-sectional view of a hybrid QD film containing 10% of Zn-CIS QDs in PbS QDs deposited onto a Si substrate by layer-by-layer spin-coating and ligand-exchange process (with ten iterations). The scale bar corresponds to 200 nm; (b) to (f) EDX mapping of Si (b), Pb (c), Cu (d), S (e) and In (f) elements on the same sample area of (a); (g) the EDX spectra detected from this area.

VI. Current-voltage (*J-V*) characteristics of TiO_2/QD bi-layer heterojunction solar cells containing different volume fraction of Zn-CIS QDs in the PbS QD layer under 3% SUN illumination

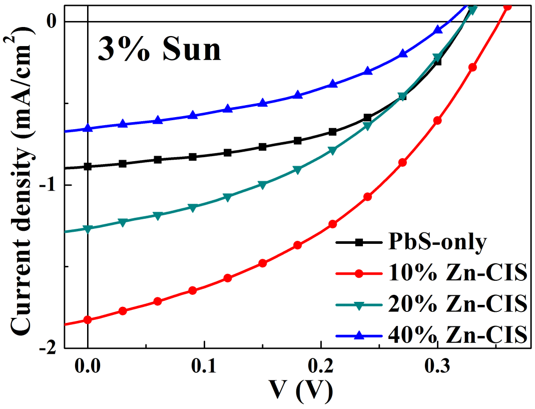

Figure S9. Current-voltage (*J-V*) characteristics of four representative TiO_2/QD bi-layer heterojunction solar cells containing 0%, 10%, 20%, and 40% of Zn-CIS QDs in the PbS QD layer under 3% SUN simulated AM1.5G illumination.

VII. Bottom-gate/bottom-contact field-effect transistor transfer characteristics of PbS QDs films containing different volume fractions of Zn-CIS QD deposited on Si/SiO_2 (300 nm) substrates.

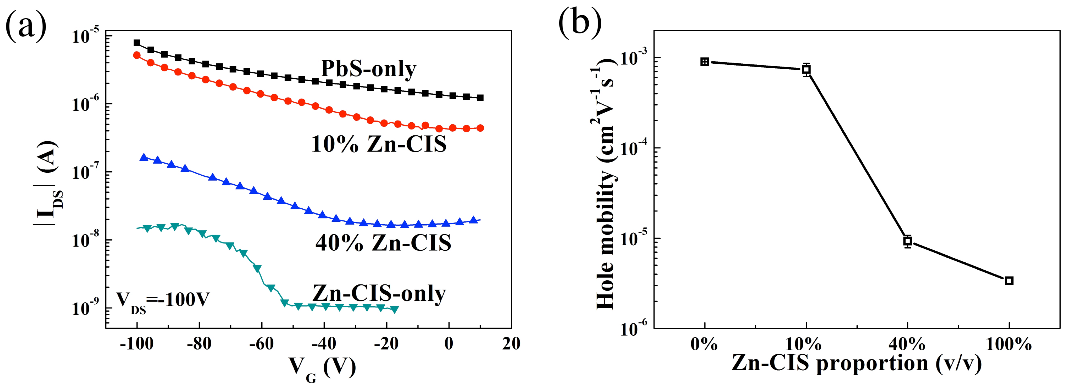

Figure S10. (a) The transfer characteristics of bottom-gate/bottom-contact thin-film field-effect transistors based on a QD film containing different volume fractions of Zn-CIS QDs in PbS QDs deposited on Si/SiO_2 (300 nm) substrates; (b) The hole mobilities extracted from the transistors shown in (a).

VIII. Experimental details on synthesis

Synthesis of nanocrystals. PbS quantum dots were synthesized by a procedure reported previously[28, 30] with a post-synthesis $CdCl_2$ halide treatment.[18] In brief, in a 50 mL three-neck flask, 18 mL of octadecene, 0.45 of g lead(II) oxide, and 1.5 mL of oleic acid were loaded and degassed under vacuum under stirring at 100 °C for the formation of lead oleate. After the lead oxide was dissolved, the mixture was kept at 125 °C under argon flow. A sulfur precursor was prepared separately inside a glovebox by mixing 10 mL of octadecene (degassed previously) and 0.18 mL of hexamethyldisilathiane. This sulfur precursor was then injected into the lead oleate solution at 125 °C. After injection, the heating was switched off and the reaction mixture was allowed to cool down to 40 °C in about 40 minutes. During this process when the temperature reached ~ 100 °C 1 ml $CdCl_2$ precursor was injected in to the PbS QD flask for halide treatment.[18] For the preparation of $CdCl_2$ precursor, 0.6 g cadmium chloride and 0.066 g tetradecylphosphonic acid were dissolved in 10 ml oleylamine under argon. This mixture was then degassed in vacuum for > 2 h at 100 °C and kept again under

argon. Post-synthesis QD precipitation procedures are identical as those published previously[28] and they were finally dispersed in anhydrous octane.

Zn-CIS QDs were synthesized according to a reported procedure.[6] Typically, 1 mmol of copper (I) iodide (190 mg), 1 mmol of indium acetate (291 mg) and 0.1 mmol of zinc acetate ($Zn(OAc)_2$, 18 mg) were introduced in a three-neck flask with 5 ml of dodecanethiol (DDT) and degassed under vacuum at 40 °C for 30 min. The yellow turbid suspension was then placed under argon flow and heated to 120 °C for 10 min. The resulting clear yellow solution was subsequently further heated to 220 °C. After approximately 15 minutes, the mixture was quickly cooled to room temperature. The QDs were then collected by precipitation with acetone and washed three times by successive redispersion and precipitation in hexane/ethanol, toluene/acetone and toluene/methanol. They are finally dispersed in anhydrous octane.
